# Adoption of Electronic Health Record Among Substance Use Disorder Treatment Programs: Nationwide Cross-Sectional Survey Study

**DOI:** 10.2196/45238

**Published:** 2023-12-14

**Authors:** Jemima A Frimpong, Xun Liu, Lingrui Liu, Ruoqiuyan Zhang

**Affiliations:** 1 New York University Abu Dhabi Abu Dhabi United Arab Emirates; 2 New York University New York, NY United States; 3 Agency for Healthcare Research and Quality US Department of Health and Human Services Rockville, MD United States

**Keywords:** adoption of technology, barriers to adoption, electronic health record, health information technology, opioid treatment programs, substance use disorder

## Abstract

**Background:**

Electronic health record (EHR) systems have been shown to be associated with improvements in care processes, quality of care, and patient outcomes. EHR also has a crucial role in the delivery of substance use disorder (SUD) treatment and is considered important for addressing SUD crises, including the opioid epidemic. However, little is known about the adoption of EHR in SUD treatment programs or the organizational-level factors associated with the adoption of EHR in SUD treatment.

**Objective:**

We examined the adoption of EHR in SUD programs, with a focus on changes in adoption from 2014 to 2017, and identified organizational-level factors associated with EHR adoption.

**Methods:**

We used data from the 2014 and 2017 National Drug Abuse Treatment System Surveys. Our analysis included 1027 SUD programs (531 in 2014 and 496 in 2017). We used chi-square and Mann-Whitney *U* tests for categorical and continuous variables, respectively, to assess changes in EHR adoption, technology use, program, and client characteristics. We also investigated differences in characteristics and barriers to adoption by EHR adoption status (adopted EHR vs had not adopted or were planning to adopt EHR). We then conducted multivariate logistic regressions to examine internal and external factors associated with EHR adoption.

**Results:**

The adoption of EHR increased significantly from 57.6% (306/531) in 2014 to 69.2% (343/496) in 2017 (*P*<.001), showing that nearly one-third (153/496, 30.8%) of SUD programs had not yet adopted an EHR system by 2017. We identified a significant increase in technology use and ownership by a parent company (*P*=.01 and *P*<.001) and a decrease in the percentage of uninsured patients in 2017 (*P*<.001), compared to 2014. Our analysis further showed significant differences by adoption status for three major barriers to adoption: (1) start-up costs, (2) ongoing financial costs, and (3) privacy or security concerns (*P*<.001). Programs that used computerized scheduling (adjusted odds ratio [AOR] 3.02, 95% CI 2.23-4.09) and billing systems (AOR 2.29, 95% CI 1.62-3.25) were more likely to adopt EHR. Similarly, ownership type, such as private nonprofit (AOR 1.86, 95% CI 1.31-2.65) and public (AOR 2.14, 95% CI 1.27-3.67), or interest in participating in a patient-centered medical home (AOR 1.93, 95% CI 1.29-2.92), were associated with an increased likelihood to adopt EHR. Overall, SUD programs were more likely to adopt an EHR system in 2017 compared to 2014 (AOR 1.44, 95% CI 1.07-1.94).

**Conclusions:**

Our findings highlighted that SUD programs may be on track to achieve widespread EHR adoption. However, there is a need for focused strategies, resources, and policies explicitly designed to systematically address barriers and tackle obstacles to expanding the adoption of EHR systems. These efforts must be holistic and address factors at multiple organizational levels.

## Introduction

Electronic health record (EHR) systems are important tools that facilitate and promote improvement in the quality and efficiency of health service delivery [1-4]. The Health Information Technology for Economic and Clinical Health Act and the American Recovery and Reinvestment Act, enacted in 2009, were established to promote the adoption of health information technology by health care organizations, including hospitals and health care providers [5,6]. The adoption of EHR has been extensively studied and analyzed, including factors associated with adoption in various health care settings, of which the vast majority were conducted in primary care and acute care, and specialized health care facilities. A small but growing body of research has examined complex health care settings, such as substance use disorder (SUD) and mental health facilities [7-11]. Existing studies have predominantly focused on usability [12,13], barriers to adoption [14], and the degree to which practitioners and other medical staff leverage advanced use cases [15,16].

There is a scarcity of research that provides a national perspective or organizational-level evidence that addresses the role of internal and external organizational factors that may influence the adoption and use of EHR in SUD programs [17,18,19]. Existing studies have however identified characteristics of Programs that offer insights into rates of adoption. The often-smaller size of treatment programs, which in turn makes start-up and maintenance of EHR costlier, has been suggested as potential explanations for the slower adoption in SUD programs [14,18]. Other factors include a lack of harmonized data elements and interoperability [20,21] and the capacity to optimize data from EHR systems [22]. Insufficient staff training and implementation strategy (eg, implementation time and planning) have also been linked with lower rates of adoption [3,23].

One study examined adoption in SUD programs and mental health facilities and reported that the levels of use in SUD programs were less common compared to those mental health facilities for both nonexclusive (mixed computer and paper) and exclusive (paper-free) EHR use. Less than 25% of both facility types reported exclusive EHR use for core clinical activities (eg, progress notes, laboratory monitoring, and prescriptions), with wide variability by ownership type, accreditation status, and across states [17]. In addition to clinical care, adoption of EHR systems in SUDs could also help address policy and cost-related constraints. For example, 42 Code of Federal Regulations [CFR] Part 2—Confidentiality of Alcohol and Drug Abuse Patient Records, has been noted as a barrier to interoperability. Yet, EHR as a tool may promote data protection, and introduce cost saving that may facilitate the implementation of evidence-based practices and improve patient outcomes [24-28]. Relatedly, a study of electronic health technology use at hospital-based SUD programs found that 68% of programs used basic EHR functionality, noting that these programs lagged behind acute care hospitals in adoption of basic EHR systems. This lag, the authors concluded, presents a missed opportunity to improve quality of care and performance [29].

Overall, there remain gaps in existing knowledge of the adoption of EHR systems in outpatient SUD treatment programs or the extent to which organizational-level characteristics are associated with adoption. This study is one of the few to explore EHR adoption in a national sample of outpatient SUD programs in the United States, and adds to the existing literature by assessing factors increasingly relevant to the adoption of EHR within a setting that is primed to benefit from technology use but is underexamined. Additionally, relevant to this study’s objective was a comprehensive assessment of potential barriers to adoption (eg, start-up costs and ongoing financial costs) and the extent to which the use of other technologies, especially for billing and scheduling, may be associated with EHR adoption. Our objective was thus twofold: (1) describe the adoption of EHR in outpatient SUD programs and changes in adoption from 2014 to 2017 and (2) identify factors associated with adoption. Our findings may offer insights into variations in adoption and correlates of adoption over time and inform strategies to reduce barriers, increase adoption, and maximize the benefits of EHR in SUD treatment.

## Methods

### Sampling Frame and Sample

We analyzed data from the National Drug Abuse Treatment Systems Survey (NDATSS), a nationally representative longitudinal survey of SUD treatment programs. A list of programs generated by the Substance Abuse and Mental Health Services Administration, which licenses treatment programs, serves as the sampling frame. SUD programs were randomly selected from the list for potential inclusion in data collection. Data were collected through telephone interviews with program directors and clinical supervisors in each program. Approximately 85% of eligible programs across the nation completed the survey across multiple waves [30]. We used the 2 recently available waves of the NDATSS, 2014 (n=531) and 2017 (n=496) [31].

### Data Collection, Reliability, and Validity

The NDATSS uses a split panel design, which is a key strength of the data. Each survey wave since the first wave included programs from previous waves (panel programs), and each wave also added representative samples of newer programs. The addition of new programs keeps the NDATSS representative of the changing population of US treatment programs and ensures adequate sample size and statistical power. The NDATSS also followed established methods that maximize reliability and validity in phone surveys, and it is further supported by related studies [25,32,33].

### Measures

#### Dependent Variable

The adoption of EHR was evaluated by two research questions: (1) whether a program has adopted EHR components (“yes” or “no”) and (2) if “no,” whether the program plans to install an EHR (“planning” or “no”). EHR components were defined as “an integrated electronic clinical information system that tracks patient health data and may include such functions as encounter notes, prescriptions, lab orders, etc.” We first categorized the EHR adoption status of a program as “yes” (the program has adopted EHR); “planning” (the program has not adopted but plans to install EHR); and “no” (the program has not adopted EHR and does not have plans to adopt). We then combined the “no” or “planning” categories in our analysis to highlight our interest in understanding factors associated with EHR adoption among treatment programs that have adopted these systems compared to those that have not.

#### Independent Variables

Based on findings from previous studies [34,35], we examined four categories of characteristics that assess the internal and external environments of programs: (1) current technology use, (2) program characteristics, (3) client characteristics, and (4) geographic location. Technology use was measured by 2 variables: use of computerized scheduling systems (0=no and 1=yes) and electronic billing systems (0=no and 1=yes). We also assessed the extent of barriers reported by programs concerning EHR adoption. We created a barriers to adoption score based on responses to 13 questions on potential barriers that impede a program from beginning or expanding the use of an EHR system (Multimedia Appendix 1 contains the full list of potential barriers to EHR adoption). Each barrier was assessed at 3 levels (not a barrier, a minor barrier, and a major barrier). We created a score for barriers to adoption by assigning the value of 1 to “not a barrier,” 2 to “minor barrier,” and 3 to “major barrier.” For each program, we calculated the barriers to adoption score by summing its score values on all 13 barriers. The barriers’ score ranged between 13 and 39. Due to small cell sizes, and for the descriptive statistics, “not a barrier” and “minor barrier” were collapsed into 1 category, “none or minor barrier,” with “major barrier” as the second category.

Program characteristics included ownership status of the program (1=private for-profit, 2=private nonprofit, and 3=public) and whether the program was owned by a hospital or mental health center (0=no and 1=yes). Programs reported whether they had signed an accountable care organization (ACO) agreement or a patient-centered medical home (PCMH) agreement (“yes” or “no”). If “no,” a total of 2 questions were asked about whether they had plans for or discussed an agreement (“yes” or “no”). For ACO or PCMH participation, programs that responded “no” to all 3 questions were categorized as 0 (not interested). Programs that have signed, planned, or discussed either an ACO or PCHM agreement were categorized as 1 (interested). Other program characteristics included program type, that is, an opioid treatment program (OTP; 0=no and 1=yes), and size of the program (assessed by the number of SUD clients). The size of the program (number of SUD clients) was recoded into a categorical variable (0-250, 250-500, 500-750, and >750). We define an OTP as a physical facility with resources dedicated specifically to treating opiate dependence with methadone or buprenorphine (excluding primary care or physician offices).

Client characteristics were assessed by the percentage of African American clients, the percentage of Hispanic clients, and the percentage of uninsured clients. Programs with a percentage of uninsured patients greater than 50% were coded as high-uninsured. Geographic location includes region (1=Northeast, 2=Southeast, 3=Midwest, and 4=Southwest) and urbanicity (0=nonurban and 1=urban) [36].

### Statistical Analysis

A total of 1352 programs completed the NDATSS in 2014 (695 programs) and 2017 (657 programs). We excluded observations with missing data and arrived at an analysis sample of 1027 programs with complete data (a total of 531 in 2014 and 496 in 2017). We first conducted bivariate analyses using the chi-square and Mann-Whitney *U* tests for categorical and continuous variables, respectively. We then assessed changes in EHR adoption, as well as internal and external organizational factors, between 2014 and 2017. Organizational factors included technology use (computerized scheduling and electronic billing), program characteristics (eg, ownership type and being a part of a PCMH), client characteristics (eg, proportion of African American and Hispanic patients and having a high level of uninsured clients), and geographic location (eg, region and urban). We also investigated differences in the characteristics of programs by EHR adoption status (adopted EHR vs had not adopted EHR or planning to adopt EHR). Next, we examined the role of barriers to adoption based on EHR adoption status. Lastly, we conducted multivariate logistic regressions, using EHR adoption status as the binary outcome and organizational factors as independent variables. We reported odds ratios and 95% CIs from the adjusted model. All of the analyses were conducted using R version 3.6 (R Core Team).

### Ethical Considerations

The New York University Abu Dhabi Institutional Review Board approved this analysis study (HRPP-2023-220) on administrative data.

## Results

### Adoption of EHR

Table 1 shows changes in the adoption of EHR from 2014 to 2017. In 2014, a total of 57.6% (306/531) of programs had adopted EHR, 22.4% (119/531) of programs were planning to adopt EHR, and 20% (106/531) of programs had not adopted EHR. By 2017, a total of 69.2% (343/496) of programs had adopted EHR, 15.7% (78/496) of programs were planning to adopt EHR, and 15.1% (75/496) of programs had not adopted EHR. Overall, the proportion of programs that had adopted EHR increased by 11.6%, while the percentage of programs that were planning to adopt EHR or those that did not have plans to adopt decreased by 6.7% and 4.9% during this period, respectively (*P*<.001).

### Characteristics of Treatment Programs, by Years

Table 1 shows the characteristics of programs in 2014 and 2017. We found significant changes in program characteristics between 2014 and 2017. The proportion of programs using computerized scheduling systems increased from 54.4% (289/531) to 62.7% (311/496; *P*=.01) and from 72.7% (386/531) to 81.5% (404/496) for electronic billing systems (*P*<.001). Compared to 2014, more programs were owned by hospitals or mental health centers in 2017 (51/531, 9.6% vs 120/496, 24.2%; *P*<.001). The number of programs with more than 50% of uninsured patients decreased significantly from 39.7% (211/531) to 22% (109/496; *P*<.001).

**Table 1:** Comparison of characteristics of substance use disorder (SUD) treatment programs in 2014 and 2017.

Characteristics					2014 (n=531)		2017 (n=496)		*P* value		
**Adoption of EHR^a^, n (%)**											<.001
	Adopted EHR			306 (57.6)		343 (69.2)					
	Planning to adopt EHR			119 (22.4)		78 (15.7)					
	Had not adopted EHR			106 (29)		75 (15.1)					
**Technology use**											
		**Computerized scheduling system, n (%)**								.01	
			No	242 (45.6)		185 (37.3)					
			Yes	289 (54.4)		311 (62.7)					
		**Electronic billing system, n (%)**								<.001	
			No	145 (27.3)		92 (18.6)					
			Yes	386 (72.7)		404 (81.5)					
		Barriers to adoption score, median (IQR)^b^		24.00 (19.00-27.00)		23.00 (18.00-27.00)		.40			
**Program characteristics**											
		**Ownership, n (%)**								.30	
			Private for-profit	127 (23.9)		130 (26.2)					
			Private nonprofit	339 (63.8)		294 (59.3)					
			Public	65 (12.2)		72 (14.5)					
		**Owned by the hospital or mental health center, n (%)**								<.001	
			No	480 (90.4)		376 (75.8)					
			Yes	51 (9.6)		120 (24.2)					
		**Part of accountable care organization, n (%)**								>.99	
			Not interested	386 (72.7)		361 (72.8)					
			Interested	145 (27.3)		135 (27.2)					
		**Part of a patient-centered medical home, n (%)**								.39	
			Not interested	388 (73.1)		375 (75.6)					
			Interested	143 (26.9)		121 (24.4)					
		**OTP^c^, n (%)**								>.99	
			Non-OTP	341 (64.2)		319 (64.3)					
			OTP	190 (35.8)		177 (35.7)					
		**OTP size (number of SUD clients), n (%)**								.44	
			0-250	219 (41.2)		186 (37.5)					
			250-500	139 (26.2)		132 (26.6)					
			500-750	66 (12.4)		59 (11.9)					
			>750	107 (20.2)		119 (24)					
**Client characteristics**											
		Percentage of African American clients, median (IQR)		9.09 (2.06-27.69)		10.00 (2.48-30.77)		.31			
		Percentage of Hispanic clients, median (IQR)		5.56 (1.47-15.67)		5.51 (1.46-16.00)		.83			
		**High uninsured, n (%)**								<.001	
			No	320 (60.3)		387 (78)					
			Yes	211 (39.7)		109 (22)					
**Geographic location**											
		**Region, n (%)**								.48	
			Northeast	151 (28.4)		135 (27.2)					
			Southeast	132 (24.9)		115 (23.2)					
			Midwest	127 (23.9)		140 (28.2)					
			Southwest	121 (22.8)		106 (21.4)					
		**Urban, n (%)**								.60	
			Not urban	345 (65)		331 (66.7)					
			Urban	186 (35)		165 (33.3)					

^a^EHR: electronic health record.

^b^For the full study sample, the minimum and maximum barrier scores ranged between 13 and 39.

^c^OTP: opioid treatment program.

### Adoption of EHR by Program Characteristics

Table 2 shows differences in characteristics for programs that had adopted EHR compared to programs that had not adopted EHR. There were significant differences by EHR adoption status on several factors. Compared to programs that had not adopted an EHR system, a greater proportion of programs that had adopted EHR used a computerized scheduling system (463/649, 71.3% vs 137/378, 36.2%; *P*<.001) or electronic billing system (557/649, 85.8% vs 233/378, 61.6%; *P*<.001). The results also showed that a greater proportion of programs that had adopted EHR were private nonprofit (410/649, 63.2% vs 223/378, 59%) or publicly owned (102/649, 15.7% vs 35/378, 9.3%), with a greater proportion of programs that had not adopted being privately owned (120/378, 31.8% vs 137/649, 21.1%). The EHR-adopted programs were commonly present with interest in participating in an ACO (198/649, 30.5% vs 82/378, 21.7%) or PCMH (196/649, 30.2% vs 68/378, 18%) and a larger program size compared to those that had not adopted an EHR system. For client characteristics, programs with a high percentage of uninsured clients reported not having adopted EHR (138/387, 36.5% vs 182/649, 28%).

**Table 2:** Characteristics of substance use disorder (SUD) treatment programs that had adopted electronic health records (EHRs), compared to programs that had not adopted EHR but plan to adopt and programs that had not adopted EHR and did not have plans to adopt.

Characteristic			No and planning (n=378)		Yes (n=649)		*P* value	
**Technology use**								
	**Computerized scheduling system, n (%)**							<.001
		No	241 (63.8)	186 (28.7)				
		Yes	137 (36.2)	463 (71.3)				
	**Electronic billing system, n (%)**							<.001
		No	145 (38.4)	92 (14.2)				
		Yes	233 (61.6)	557 (85.8)				
	Barriers to adoption score, median (IQR)^a^		24.0 (19.00-27.75)	23.0 (18.00-27.00)		.05		
**Program characteristics**								
	**O** **wnership, n (%)**							<.001
		Private for-profit	120 (31.8)	137 (21.1)				
		Private nonprofit	223 (59.0)	410 (63.2)				
		Public	35 (9.3)	102 (15.7)				
	**O** **wned by the hospital or mental health center, n (%)**							.07
		No	326 (86.2)	530 (81.7)				
		Yes	52 (13.8)	119 (18.3)				
	**Part of accountable care organization, n (%)**							.003
		Not interested	296 (78.3)	451 (69.5)				
		Interested	82 (21.7)	198 (30.5)				
	**Part of a patient-centered medical home, n (%)**							<.001
		Not interested	310 (82.0)	453 (69.8)				
		Interested	68 (18.0)	196 (30.2)				
	**OTP^b^, n (%)**							.07
		Non-OTP	257 (68.0)	403 (62.1)				
		OTP	121 (32.0)	246 (37.9)				
	**OTP size (number of SUD clients), n (%) **							<.001
		0-250	182 (48.2)	223 (34.4)				
		250-500	91 (24.1)	180 (27.7)				
		500-750	39 (10.3)	86 (13.3)				
		>750	66 (17.5)	160 (24.7)				
**Client characteristics**								
	Percentage of African American clients, median (IQR)		9.6 (2.41-27.24)	9.7 (2.25-30.00)		.76		
	Percentage of Hispanic clients, median (IQR)		6.3 (1.38-16.62)	5.0 (1.57-15.38)		.52		
	**High uninsured, n (%)**							.006
		No	240 (63.5)	467 (72.0)				
		Yes	138 (36.5)	182 (28.0)				
**Geographic location**								
	**Region, n (%)**							.007
		Northeast	129 (34.1)	157 (24.2)				
		Southeast	82 (21.7)	165 (25.4)				
		Midwest	93 (24.6)	174 (26.8)				
		Southwest	74 (19.6)	153 (23.6)				
	**Urban, n (%)**							.04
		Not urban	233 (61.6 %)	443 (68.3 %)				
		Urban	145 (38.4 %)	206 (31.7 %)				

^a^For the full study sample, the minimum and maximum barrier scores ranged between 13 and 39.

^b^OTP: opioid treatment program.

### Barriers to EHR Adoption

**Figure 1:**
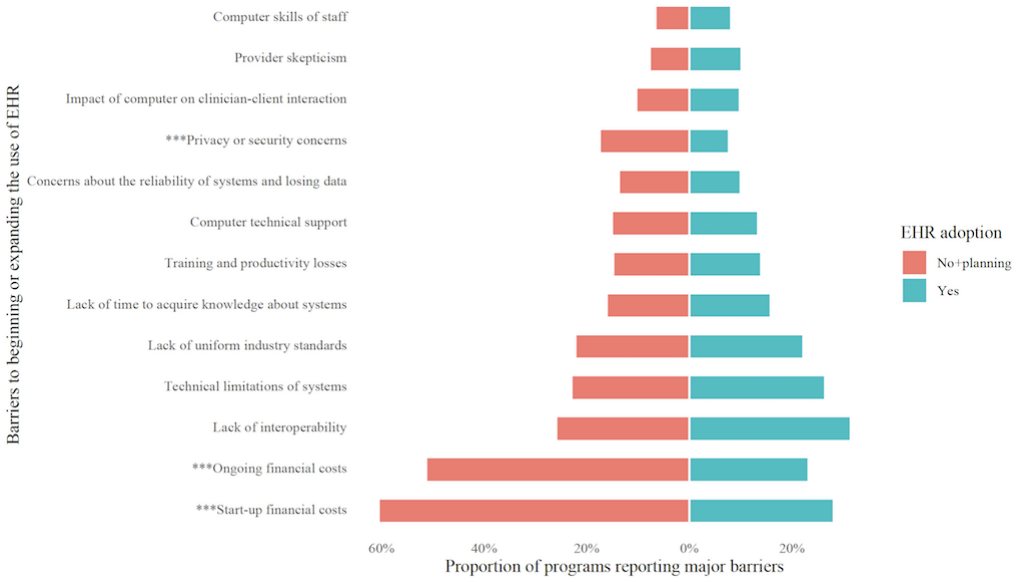
Major barriers to the adoption of electronic health record (EHR) among programs that had adopted EHR, compared with programs that had not adopted EHR but plan to adopt, and programs that had not adopted EHR and did not have plans to adopt. ***P<.001.

Figure 1 reports differences in barriers to beginning or expanding the use of EHR reported by programs that had adopted a system versus those that had not adopted. We show the proportions of programs that report each type as a major barrier. We identified that the top 5 major barriers for all programs were the same, regardless of adoption status. Specifically, programs reported that start-up costs, ongoing financial costs, a lack of interoperability, technical limitations (eg, systems either too complex or too simple to meet their needs), and a lack of uniform industry standards were major barriers. However, the proportion of programs reporting these barriers varied in rank order. Programs that had not adopted EHR reported the following percentages for each of the aforementioned barriers: start-up costs (229/378, 60.6%), ongoing financial costs (194/378, 51.3%), lack of interoperability (98/378, 25.9%), technical limitations (87/378, 23.03%), and lack of uniform industry standards (84/378, 22.2%), compared to the following percentages for programs that had adopted an EHR system: start-up costs (183/649, 28.2%), ongoing financial costs (151/649, 23.3%), lack of interoperability (204/649, 31.4%), technical limitations (172/649, 26.5%), and lack of uniform industry standards (144/649, 22.2%). In our bivariate analysis, stratified by year, we found that among the 13 barriers, start-up costs, ongoing financial costs, and privacy or security concerns were significantly associated with EHR adoption status (*P*<.001).

### Factors Associated With EHR Adoption

**Figure 2:**
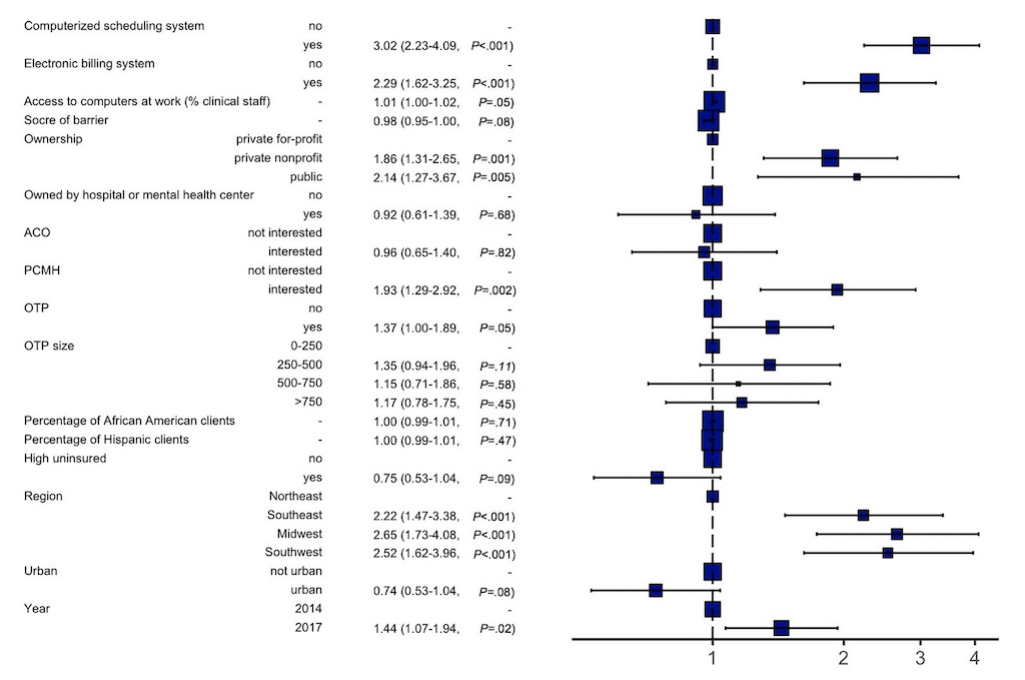
Multivariate logistic regression results for the adoption of electronic health records (EHRs) among programs that had adopted EHR, compared with programs that had not adopted EHR but plan to adopt and programs that had not adopted EHR and did not have plans to adopt. ACO: accountable care organization; OTP: opioid treatment program; PCMH: patient-centered medical home.

Figure 2 presents results from the adjusted logistic regression model. Programs with a computerized scheduling system (adjusted odds ratio [AOR] 3.05, 95% CI 2.25-4.13) or an electronic billing system (AOR 2.33, 95% CI 1.64-3.30) were more likely to adopt EHR. Compared to private for-profit programs, private nonprofit (AOR 1.91, 95% CI 1.34-2.71) and public (AOR 2.27, 95% CI 1.35-3.88) programs reported a greater likelihood of having adopted EHR. We also found that adoption of EHR was positively associated with participation in a PCMH agreement (AOR 1.96, 95% CI 1.31-2.97). Our results indicated regional differences in adoption, with programs in the Midwest having the highest EHR adoption (AOR 2.66, 95% CI 1.74-4.10). SUD programs were more likely to adopt an EHR system in 2017, compared to 2014 (AOR 1.45, 95% CI 1.08-1.95).

## Discussion

### Overview

Using a nationally representative US sample of SUD treatment programs, we conducted one of few studies, to the best of our knowledge, on the adoption of EHR in stand-alone outpatient SUD programs. This study demonstrated a robust examination of differences in EHR adoption in SUD treatment programs over the interval between 2014 and 2017 and characterized the internal and external organizational factors associated with EHR adoption.

### Patterns of EHR Adoption and Organizational Characteristics

Descriptive results showed that the adoption of EHR in the context of SUD treatment had significantly increased, by nearly 12%, from 2014 to 2017. However, 30.8% (153/496) of SUD treatment programs had not adopted EHR by 2017. Our finding is in line with previous studies that reported that the adoption and use of EHR are significantly less common in SUD programs compared to other health care facilities, including mental health treatment centers and hospitals, and highlighted the need for further efforts to increase adoption in SUD programs [17,29,34]. One study reported that although use of any EHR functionality (eg, treatment planning and prescription) rangedfrom 80% to 95% in SUD programs, exclusive EHR use of any functionality (a paper-free system) ranged from 26% to 48% [17]. These findings are relevant for this study and suggest that among the SUD programs that reported adopting EHR, the majority may not be maximizing the benefits of EHR functionalities and systems. We also found significant differences by technology use, patient, client, and geographic location based on EHR adoption status. Specifically, use of computerized scheduling, electronic billing, and ownership by a hospital or mental health center increased significantly from 2014 to 2017, with a decrease in the proportion of uninsured patients during the same period [14,35].

### Internal and External Organizational Factors Associated With EHR Adoption

Our adjusted model showed that multiple SUD treatment program internal and external organizational characteristics were positively associated with a program adopting an EHR system. Program technology use capabilities, including the program’s computerized scheduling system and electronic billing system, were significantly associated with EHR adoption. We posit that perhaps a program that has already implemented a computerized scheduling system and an electronic billing system may have a higher propensity or advanced level of knowledge that could be foundational and facilitate the adoption of an EHR system [34]. A greater technology use capability (eg, a computerized scheduling system and an electronic billing system) could also indicate that the organizational culture and context would be more supportive of using the EHR system [37,38]. For example, organizational culture has been shown to moderate the relationship between attitudes toward EHR and intentions to adopt EHR [39]. This could also signal that leadership and health care administrators could be more likely to test varying systems and approaches to care [40]. These leaders may thus have a more positive perception of the advantages of using health information technology to improve efficiency and provide high-quality and safer treatment services to patients [41].

An incremental approach to deploying EHR (eg, the initial introduction of computerized systems for scheduling or billing) may also be relevant. Health care providers and staff may be more likely to use the EHR system and have a more positive perception of its use when integrated into the workflow [39,42,43]. These findings suggest that, rather than implementing a comprehensive EHR system in SUD treatment programs in a single phase, program managers should consider introducing EHR in multiple phases. The integrated and workflow-focused approach is particularly useful in health systems that manage complex patients and necessarily require coordination between providers or across health care facilities (eg, integrating SUD treatment in primary care) [21]. This approach, along with an organizational culture where staff are trained in EHR functionalities related to their core practice areas, may further EHR adoption [17,18]. This adoption strategy highlights key organizational factors that have been previously identified and could be intervened upon to incentivize EHR adoption and implementation.

We found that programs that were public or private, of nonprofit ownership, or interested in participating in a PCMH were more likely to adopt EHR. Most of the research that has considered ownership type has explored hospital-based specialty SUD treatment programs [44,45], but some have focused on SUD facilities [46,47]. The studies often report that facilities with nonprofit ownership are more likely to adopt EHR systems compared to for-profit facilities. Compared to nonprofit hospitals, for-profit and government-owned facilities had less likely odds of basic EHR adoption [29]. Exclusive use of EHR for core clinical purposes has also been associated with public ownership among SUD facilities, with nonprofit ownership compared to private for-profit ownership [17]. It is worth noting that findings on public ownership and EHR adoption, especially in behavioral and SUD contexts, have been mixed. Some studies show that private facilities have higher rates of EHR adoption [48], while others show that public and nonprofit facilities are more likely to adopt EHR [49]. These findings generate further questions on the differences in adoption among different systems and whether structural factors, such as ownership type, may necessitate varied implementation strategies. and the extent to which the allocation of resources and incentives may promote adoption and use.

Existing research has also broadly demonstrated the relationship between the use of health information technology, including EHRs, and participation in incentive-based programs such as PCMHs [29]. Most of the studies of PCMHs have, however, focused on ambulatory health care, long-term care facilities, and hospitals [50-53]. Less attention has been given to SUD programs and has thus created gaps in knowledge on how these initiatives influence service delivery in the SUD context [46,47,54]. Additionally, achieving PCMH status is intricately linked with health information technologies and is equally relevant to SUD programs. Specifically, the National Committee for Quality Assurance, in its introduction of the PCMH framework, noted that technologies, including EHR, to support the documentation and tracking of care are aligned with improving quality of care [55]. Accordingly, EHR systems, through care coordination, exchange of health data, and data use, have been shown to provide a pathway to streamline the implementation of PCMH [56]. Strategies to promote and facilitate the adoption of EHR in SUD programs should consider models such as participation in a PCMH, which may serve as catalysts for adoption. These care models are designed to promote information sharing, coordination, and collaboration across providers and are well aligned with the broader objectives of care integration.

Integrating mental health and behavioral health services programs into comprehensive primary health care, for example, has been widely acknowledged as crucial to addressing behavioral health crises [57,58]. The implementation and use of evidence-based practices to facilitate integrated behavioral health has become an urgent policy concern and a priority in reforming health systems in the United States [59,60]. Of the identified major obstacles in integrated behavioral health care, how to effectively address the fragmentation of SUD treatment facilities from larger health systems (eg, the excluded health information exchange systems and billing systems) has been considered critical. In particular, SUD treatment services have often been provided in separate specialty SUD programs or mental health programs. SUD treatment data similarly tend to be excluded from the EHRs of larger health systems [61,62]. This fragmented service delivery persists, even though SUD patients often require interdisciplinary care from a diverse range of specialists and health care providers across different health care institutions. EHRs have the potential to facilitate these processes by enhancing communication and promoting care coordination. These efforts, if successful, could have an overall positive impact on improving care delivery, including adherence to guidelines, reductions in medication errors, and the cost of care [26,57].

The cost of EHR has been noted as one of the largest contributing factors to the low rates of EHR adoption [63]. We identified 3 major barriers among the 13 barriers to adoption, including start-up financial costs, ongoing financial costs, and privacy or security concerns.

An earlier study investigated the long-standing cost-related barriers to the adoption and use of EHR and found that they may be associated with the uncertain determination of the health care entity that should bear these costs. Specifically, while health care payers may realize the most benefit, health care organizations that deliver services often finance EHR adoption and implementation. These costs extend beyond the initial uptake of these systems, with additional costs incurred as a result of system upgrades and maintenance. This payment-benefit disconnect, with respect to EHR systems, creates a disincentive for health care delivery organizations to add EHR systems or adopt a comprehensive set of functionalities to their operations [63]. Interoperability of EHR among programs due to privacy and security restrictions by the federal regulatory 42 CFR Part 2 has also been shown to be a common barrier to EHR adoption [23,27,33]. These constraints are compounded by SUD treatment programs, which are often smaller in size and subject to even more funding constraints [17,64].

Understanding changes in the adoption of EHR and factors associated with adoption over time is an important first step to developing strategies and policies that promote and facilitate the adoption of EHR in SUD programs. This paper has identified the pattern of EHR adoption as well as the characteristics of treatment programs that may have influenced the adoption of EHR between 2014 and 2017. Our research identified program characteristics that could be intervened upon and provide incentives for SUD treatment programs to implement EHR. These efforts should emphasize the multiple organizational tensions of innovation processes, for example, adoption and use of EHR, and highlight the relevance of research and policy-informed management of organizations’ tensions as a key strategic direction [43].

### Limitations

There are some limitations to our study that must be considered. The latest year of data included in our analysis is 2017 [42], and there may have been considerable changes in the adoption of EHR in SUD programs since this period. In addition, the NDATSS is cross-sectional, and we therefore cannot examine causal relationships. This study, however, remains relevant in that it is one of the few studies to date to examine the adoption of EHR in outpatient SUD programs. Our findings highlighted patterns of EHR adoption in SUD treatment and organizational-level factors associated with adoption. This study also makes an important contribution to knowledge of SUD programs and EHR adoption and provides information on a robust set of factors that may have implications for policy and practice. Future studies, based on more recent data, are needed to further support our findings. These studies should aim to identify changes in adoption rates and examine changes in SUD operating environments that may influence patterns of EHR adoption and use.

Moreover, we did not examine the features of EHR systems adopted by programs or the use of EHR features. This level of analysis would provide additional information that may be useful for understanding the relationship between features and the use of EHR systems. These analyses could also provide further insights into how the adoption of EHR may influence other outcomes, especially those at the patient level. Lastly, SUD treatment programs are dynamic and operate in a policy-intensive and resource-constrained environment. Future studies should therefore include a focus on policies, processes, and sources of funding.

### Conclusions

Notwithstanding the limitations of this study, our findings contribute to addressing gaps in an organizational perspective on EHR adoption in outpatient SUD programs and do so from a national perspective. Our results show that the adoption of EHR in SUD treatment programs increased from 2014 to 2017, yet a considerable proportion of programs had not adopted an EHR system by 2017. We identified barriers to EHR adoption and associations between adoption of an EHR and key characteristics of programs, including previous use of other technology, ownership type, and interest in participating in a PCMH. One of the salient hurdles, unveiled by our research, is the persistent cost and financing related to EHR adoption. Multipronged strategies that promote EHR adoptions, including incentive models and processes that address the identified barriers, are needed to reinforce internal and external organization characteristics that are likely to facilitate the adoption and use of EHR in SUD programs.

## Appendix

Multimedia Appendix 1 Potential barriers to the adoption of electronic health records in substance use disorder treatment programs.

## Abbreviations

ACO: accountable care organization
AOR: adjusted odds ratio
CFR: Code of Federal Regulations
EHR: electronic health record
HITECH: Health Information Technology for Economic and Clinical Health
NDATSS: National Drug Abuse Treatment System Survey
OTP: opioid treatment program
PCMH: patient-centered medical home
SUD: substance use disorder

## Data Availability

The data sets analyzed during the current study are publicly available from the National Addiction and & HIV Data Archive Program.

## References

[1] Atasoy H, Greenwood BN, McCullough JS (2019). The digitization of patient care: a review of the effects of electronic health records on health care quality and utilization. Annu Rev Public Health.

[2] Coffey RM, Buck JA, Kassed CA, Dilonardo J, Forhan C, Marder WD, Vandivort-Warren R (2008). Transforming mental health and substance abuse data systems in the United States. Psychiatr Serv.

[3] Kruse CS, Beane A (2018). Health information technology continues to show positive effect on medical outcomes: systematic review. J Med Internet Res.

[4] Lin HL, Wu DC, Cheng SM, Chen CJ, Wang MC, Cheng CA (2020). Association between electronic medical records and healthcare quality. Medicine (Baltimore).

[5] Buntin MB, Burke MF, Hoaglin MC, Blumenthal D (2011). The benefits of health information technology: a review of the recent literature shows predominantly positive results. Health Aff (Millwood).

[6] Steinbrook R (2009). Health care and the American recovery and reinvestment act. N Engl J Med.

[7] Adler-Milstein J, Everson J, Lee SYD (2015). EHR adoption and hospital performance: time-related effects. Health Serv Res.

[8] Charles D, Gabriel M, Furukawa MF (2013). Adoption of electronic health record systems among US non-federal acute care hospitals. ONC data brief.

[9] Frimpong JA, Jackson BE, Stewart LM, Singh KP, Rivers PA, Bae S (2013). Health information technology capacity at federally qualified health centers: a mechanism for improving quality of care. BMC Health Serv Res.

[10] Jones EB, Furukawa MF (2014). Adoption and use of electronic health records among federally qualified health centers grew substantially during 2010-12. Health Aff (Millwood).

[11] Spatar D, Kok O, Basoglu N, Daim T (2019). Adoption factors of electronic health record systems. Technol Soc.

[12] Dentzer S (2010). One year after the stimulus, will we get health IT right?. Health Aff.

[13] Kutney-Lee A, Sloane DM, Bowles KH, Burns LR, Aiken LH (2019). Electronic health record adoption and nurse reports of usability and quality of care: the role of work environment. Appl Clin Inform.

[14] Kruse CS, Kothman K, Anerobi K, Abanaka L (2016). Adoption factors of the electronic health record: a systematic review. JMIR Med Inform.

[15] Adler-Milstein J, Holmgren AJ, Kralovec P, Worzala C, Searcy T, Patel V (2017). Electronic health record adoption in US hospitals: the emergence of a digital "advanced use" divide. J Am Med Inform Assoc.

[16] Mekhjian HS, Kumar RR, Kuehn L, Bentley TD, Teater P, Thomas A, Payne B, Ahmad A (2002). Immediate benefits realized following implementation of physician order entry at an academic medical center. J Am Med Inform Assoc.

[17] Spivak S, Strain EC, Cullen B, Ruble AAE, Antoine DG, Mojtabai R (2021). Electronic health record adoption among US substance use disorder and other mental health treatment facilities. Drug Alcohol Depend.

[18] Wisdom JP, Ford JH, McCarty D (2010). The use of health information technology in publicly-funded U. S. substance abuse treatment agencies. Contemp Drug Probl.

[19] Jung SY, Hwang H, Lee K, Lee D, Yoo S, Lim K, Lee HY, Kim E (2021). User perspectives on barriers and facilitators to the implementation of electronic health records in behavioral hospitals: qualitative study. JMIR Form Res.

[20] Ghitza UE, Gore-Langton RE, Lindblad R, Shide D, Subramaniam G, Tai B (2013). Common data elements for substance use disorders in electronic health records: the NIDA clinical trials network experience. Addiction.

[21] Tai B, McLellan AT (2012). Integrating information on substance use disorders into electronic health record systems. J Subst Abuse Treat.

[22] Wisdom JP, Ford JH, Wise M, Mackey D, Green CA (2011). Substance abuse treatment programs' data management capacity: an exploratory study. J Behav Health Serv Res.

[23] Gold R, Bunce A, Cowburn S, Dambrun K, Dearing M, Middendorf M, Mossman N, Hollombe C, Mahr P, Melgar G, Davis J, Gottlieb L, Cottrell E (2018). Adoption of social determinants of health EHR tools by Community Health Centers. Ann Fam Med.

[24] Campbell ANC, McCarty D, Rieckmann T, McNeely J, Rotrosen J, Wu LT, Bart G (2019). Interpretation and integration of the federal substance use privacy protection rule in integrated health systems: a qualitative analysis. J Subst Abuse Treat.

[25] Frimpong JA, D'Aunno T, Helleringer S, Metsch LR (2016). Low rates of adoption and implementation of rapid HIV testing in substance use disorder treatment programs. J Subst Abuse Treat.

[26] Ghitza UE, Sparenborg S, Tai B (2011). Improving drug abuse treatment delivery through adoption of harmonized electronic health record systems. Subst Abuse Rehabil.

[27] Guerrero EG, Frimpong J, Kong Y, Fenwick K, Aarons GA (2020). Advancing theory on the multilevel role of leadership in the implementation of evidence-based health care practices. Health Care Manage Rev.

[28] Henry J, Pylypchuk Y, Searcy T, Patel V (2016). Adoption of electronic health record systems among US non-federal acute care hospitals: 2008–2015. ONC data brief.

[29] Shields MC, Horgan CM, Ritter GA, Busch AB (2021). Use of electronic health information technology in a national sample of hospitals that provide specialty substance use care. Psychiatr Serv.

[30] Chen Q, Wilson DM, D'Aunno T (2017). National Drug Abuse Treatment System Survey (NDATSS): Sampling and Weighting Documentation for NDATSS 2016-2017.

[31] D'Aunno TA, Friedmann PD (2023). National drug abuse treatment system survey, waves V-IX, [United States], 2000-2017 (ICPSR 38420). National Addiction and HIV Data Archive Program: Inter-university Consortium for Political and Social Research.

[32] D'Aunno T, Park SE, Pollack HA (2019). Evidence-based treatment for opioid use disorders: a national study of methadone dose levels, 2011-2017. J Subst Abuse Treat.

[33] Groves RM, Presser S, Tourangeau R, West BT, Couper MP, Singer E, Toppe C (2012). Support for the survey sponsor and nonresponse bias. Public Opin Q.

[34] Ducharme LJ, Knudsen HK, Roman PM (2005). Computer systems in addiction treatment programs: availability and implications for program evaluation. Eval Program Plan.

[35] Saunders H, Rudowitz R (2022). Demographics and health insurance coverage of nonelderly adults with mental illness and substance use disorders in 2020. Kaiser Family Foundation.

[36] Ingram DD, Franco SJ (2012). NCHS urban-rural classification scheme for counties. Vital Health Stat 2.

[37] Li SA, Jeffs L, Barwick M, Stevens B (2018). Organizational contextual features that influence the implementation of evidence-based practices across healthcare settings: a systematic integrative review. Syst Rev.

[38] Rajamani S, Hultman G, Bakker C, Melton GB (2022). The role of organizational culture in health information technology implementations: a scoping review. Learn Health Syst.

[39] Jianxun C, Arkorful VE, Shuliang Z (2021). Electronic health records adoption: do institutional pressures and organizational culture matter?. Technol Soc.

[40] Palabindala V, Pamarthy A, Jonnalagadda NR (2016). Adoption of electronic health records and barriers. J Community Hosp Intern Med Perspect.

[41] Esdar M, Hübner U, Thye J, Babitsch B, Liebe JD (2021). The effect of innovation capabilities of health care organizations on the quality of health information technology: model development with cross-sectional data. JMIR Med Inform.

[42] Hsiao CJ, Jha AK, King J, Patel V, Furukawa MF, Mostashari F (2013). Office-based physicians are responding to incentives and assistance by adopting and using electronic health records. Health Aff (Millwood).

[43] Haring M, Freigang F, Amelung V, Gersch M (2022). What can healthcare systems learn from looking at tensions in innovation processes? a systematic literature review. BMC Health Serv Res.

[44] Furukawa MF, Raghu TS, Spaulding TJ, Vinze A (2008). Adoption of health information technology for medication safety in U.S. hospitals, 2006. Health Aff (Millwood).

[45] Rodriguez HP, McClellan SR, Bibi S, Casalino LP, Ramsay PP, Shortell SM (2016). Increased use of care management processes and expanded health information technology functions by practice ownership and medicaid revenue. Med Care Res Rev.

[46] D'Aunno T, Pollack H, Chen Q, Friedmann PD (2017). Linkages between patient-centered medical homes and addiction treatment organizations: results from a national survey. Med Care.

[47] Park SE, Mosley JE, Grogan CM, Pollack HA, Humphreys K, D'Aunno T, Friedmann PD (2020). Patient-centered care's relationship with substance use disorder treatment utilization. J Subst Abuse Treat.

[48] Adler-Milstein J, Jha AK (2017). HITECH act drove large gains in hospital electronic health record adoption. Health Aff (Millwood).

[49] Hu X, Qu H, Houser SH, Chen H, Zhou J, Yu M (2020). Hospital characteristics associated with certified EHR adoption among US psychiatric hospitals. Risk Manag Healthc Policy.

[50] Kern LM, Edwards A, Kaushal R (2014). The patient-centered medical home, electronic health records, and quality of care. Ann Intern Med.

[51] Kraschnewski JL, Gabbay RA (2013). Role of health information technologies in the patient-centered medical home. J Diabetes Sci Technol.

[52] Leventhal T, Taliaferro JP, Wong K, Hughes C, Mun S (2012). The patient-centered medical home and health information technology. Telemed J E Health.

[53] Meyers D, Quinn M, Clancy CM (2011). Health information technology: turning the patient-centered medical home from concept to reality. Am J Med Qual.

[54] Balio CP, Apathy NC, Danek RL (2019). Health information technology and accountable care organizations: a systematic review and future directions. EGEMS (Wash DC).

[55] (2011). Standards and guidelines for NCQA's Patient-Centered Medical Home (PCMH) 2011. Assurance NCfQ.

[56] Gelmon S, Bouranis N, Sandberg B, Petchel S (2018). Strategies for addressing the challenges of patient-centered medical home implementation: lessons from oregon. J Am Board Fam Med.

[57] Adeniran E, Quinn M, Wallace R, Walden RR, Labisi T, Olaniyan A, Brooks B, Pack R (2023). A scoping review of barriers and facilitators to the integration of substance use treatment services into US mainstream health care. Drug Alcohol Depend Rep.

[58] Brousselle A, Lamothe L, Sylvain C, Foro A, Perreault M (2010). Integrating services for patients with mental and substance use disorders: what matters?. Health Care Manage Rev.

[59] Jemberie WB, Williams JS, Eriksson M, Grönlund AS, Ng N, Nilsson MB, Padyab M, Priest KC, Sandlund M, Snellman F, McCarty D, Lundgren LM (2020). Substance use disorders and COVID-19: multi-faceted problems which require multi-pronged solutions. Front Psychiatry.

[60] Volkow ND, Blanco C (2021). Research on substance use disorders during the COVID-19 pandemic. J Subst Abuse Treat.

[61] McLellan AT, Woodworth AM (2014). The affordable care act and treatment for "substance use disorders:" implications of ending segregated behavioral healthcare. J Subst Abuse Treat.

[62] Wu LT, Payne EH, Roseman K, Case A, Nelson C, Lindblad R (2020). Using a health information technology survey to explore the availability of addiction treatment data in the electronic health records: a national drug abuse treatment clinical trials network study. J Subst Abuse Treat.

[63] Sittig DF, Singh H (2011). Legal, ethical, and financial dilemmas in electronic health record adoption and use. Pediatrics.

[64] Guerrero EG, Amaro H, Kong Y, Khachikian T, Marsh JC (2023). Understanding the role of financial capacity in the delivery of opioid use disorder treatment. BMC Health Serv Res.

