# Supplementary material for: Adoption of Electronic Health Record Among Substance Use Disorder Treatment Programs: Nationwide Cross-Sectional Survey Study
Source: J Med Internet Res. 2023 Dec 14;25:e45238. doi: 10.2196/45238 (PMC10755658; doi:10.2196/45238)
Supplement: Multimedia Appendix 1 [file jmir_v25i1e45238_app1.docx]

| 1 | Computer skills of staff |
| --- | --- |
| 2 | Computer technical support |
| 3 | Lack of time to acquire knowledge about systems |
| 4 | Concerns about the reliability of systems and losing data |
| 5 | Start-up financial costs |
| 6 | Ongoing financial costs |
| 7 | Training and productivity losses |
| 8 | Provider skepticism |
| 9 | Privacy or security concerns |
| 10 | Impact of computer on clinician-client interaction (i.e., rapport) |
| 11 | Lack of uniform industry standards (i.e., vendor products differ widely) |
| 12 | Technical limitations of systems (e.g., systems either too complex or too simple to meet our needs) |
| 13 | Lack of interoperability (i.e., inability of different systems to communicate) |
